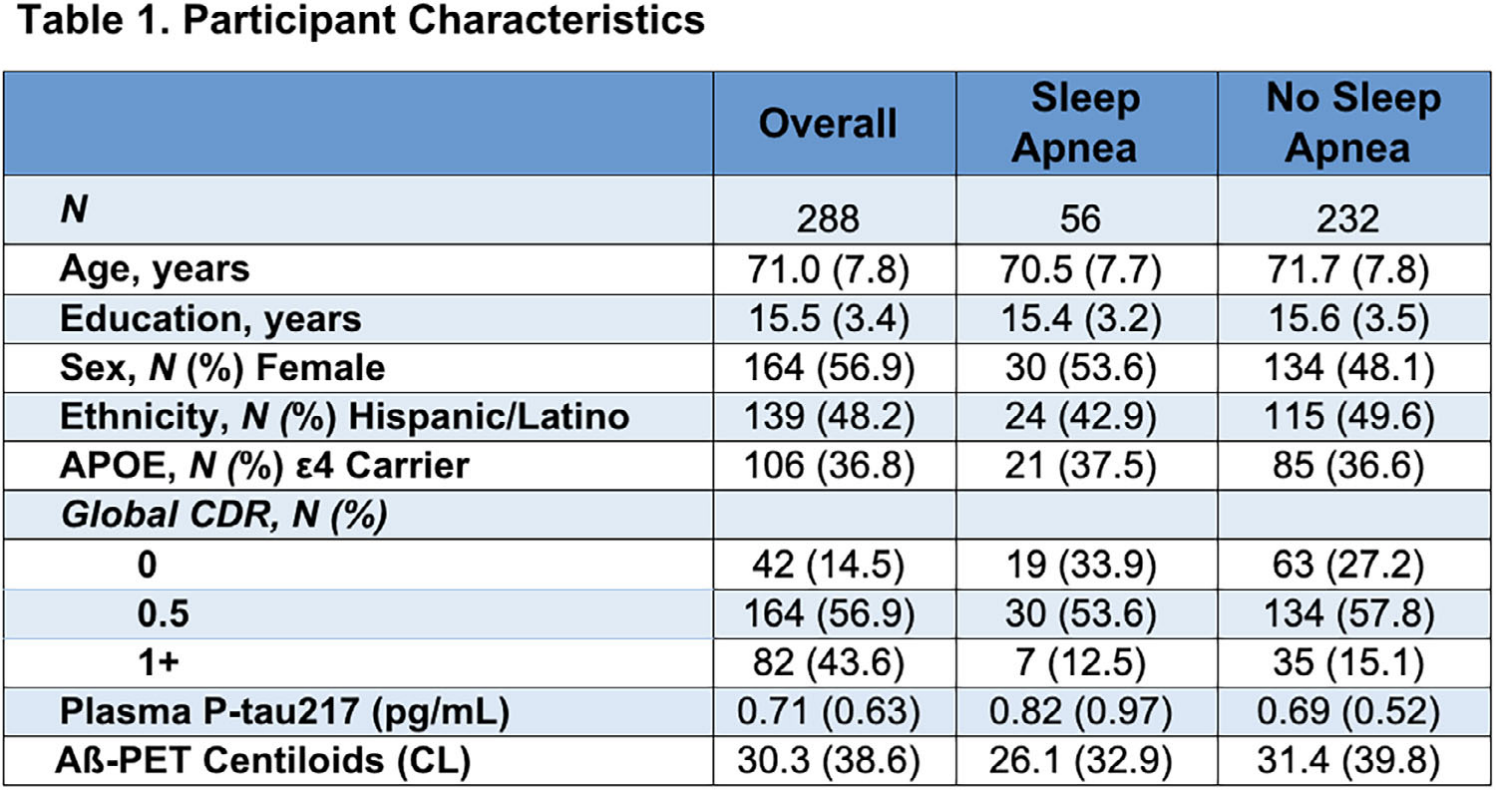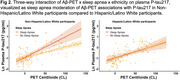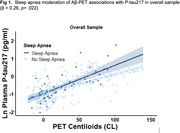# Sleep apnea moderation of cortical amyloid‐β and plasma *p*‐tau217 in ethnically diverse older adults

**DOI:** 10.1002/alz70856_107710

**Published:** 2026-01-09

**Authors:** Shannon Y. Lee, Olivia M Emanuel, Emily F Matusz, Wei‐en Wang, Franchesca Arias, Shellie‐Anne Levy, Idaly Velez‐Uribe, Warren W Barker, Monica Rosselli, David E. Vaillancourt, Melissa J. Armstrong, Rosie E Curiel Cid, David A. Loewenstein, Ranjan Duara, Glenn E. Smith, Breton M. Asken

**Affiliations:** ^1^ University of Florida, Gainesville, FL, USA; ^2^ 1Florida Alzheimer's Disease Research Center, Miami, FL, USA; ^3^ Florida Atlantic University, Davie, FL, USA; ^4^ Florida Alzheimer's Disease Research Center, Gainesville, FL, USA; ^5^ Mount Sinai Medical Center, Miami Beach, FL, USA; ^6^ 1Florida Alzheimer's Disease Research Center, Department of Clinical and Health Psychology, University of Florida, Gainesville, FL, USA; ^7^ 1Florida Alzheimer's Disease Research Center, Gainesville, FL, USA

## Abstract

**Background:**

Sleep apnea is a potential risk factor for Alzheimer's Disease (AD). Associations between sleep apnea and elevated AD biomarkers like amyloid beta (Aβ) and *p*‐tau have been reported, but it is unclear if or how sleep apnea influences the connection between the two. The link between sleep apnea and AD also has not been extensively studied in the context of relevant demographic, sociocultural, and common genetic factors. Therefore, we assessed the moderating effects of sleep apnea on the association between Aβ‐PET and plasma *p*‐tau217 and whether this moderation differed based on sex, ethnicity, or *APOE* e4 carrier status.

**Method:**

We studied 1Florida ADRC participants (*N* = 288) with normal cognition, mild cognitive impairment, or dementia (Table 1). Presence or absence of sleep apnea was determined from the National Alzheimer's Coordinating Center Health History. All participants had plasma samples analyzed for *p*‐tau217 (ALZPath) and completed Aβ‐PET with [18F] florbetaben or florbetapir. Global standardized uptake value ratio (SUVR; whole cerebellum reference) was calculated and converted to the Centiloid (CL) scale. We used multiple linear regression to assess the interaction of Aβ‐PET and sleep apnea status on plasma *p*‐tau217, controlling for age, sex, and CDR sum of boxes. To determine whether sleep apnea moderator effects differed by *APOE* e4 carrier status, sex, or ethnicity (Hispanic/Latino vs. non‐Hispanic/Latino), we employed three‐way interactions.

**Result:**

Sleep apnea moderated Aβ‐PET associations with *p*‐tau217 (β = 0.26, *p* = .022; Figure 1), such that greater amyloid burden related more strongly to higher plasma *p*‐tau217 in those with sleep apnea versus without. A significant three‐way interaction of Aβ‐PET x sleep apnea x ethnicity on plasma *p*‐tau217 (β = ‐0.52, *p* = .024; Figure 2) revealed that sleep apnea only moderated this association in non‐Hispanic/Latino participants. Sleep apnea moderation was not dependent on sex or *APOE* e4 carrier status.

**Conclusion:**

Addressing sleep apnea as a modifiable risk factor may promote slowing or resistance to AD. Larger and longitudinal studies are needed to comprehensively examine sleep apnea in older adults. Exploring sleep apnea effects in more representative samples with consideration of social and structural determinants of brain health will help clarify the role of sleep on AD onset and progression.